# The role of the Field Epidemiology Training Program in the public health emergency response: Sudan armed conflict 2023

**DOI:** 10.3389/fpubh.2024.1300084

**Published:** 2024-01-31

**Authors:** Amna Khairy, Haitham Bashier, Hatim Nuh, Nagla Ahmed, Yousif Ali, Ahmad Izzoddeen, Sara Mohamed, Muntasir Osman, Yousef Khader

**Affiliations:** ^1^Sudan Field Epidemiology Training Program, Khartoum, Sudan; ^2^Global Health Development/Eastern Mediterranean Public Health Network, Amman, Jordan; ^3^Remote Sensing Authority, National Center for Research, Khartoum, Sudan; ^4^Department of Public Health, Jordan University of Science and Technology, Irbid, Jordan

**Keywords:** armed conflict, field epidemiology, crisis response, Africa, health security

## Abstract

**Background:**

On April 15, 2023, the armed conflict between the Sudanese Armed Forces (SAF) and the Rapid Support Forces (RSF) started in Khartoum state, Sudan. This conflict was complicated by the preexisting complicated epidemiological situation and fragile health system in Sudan. This study endeavors to illuminate the pivotal role essayed by the Sudan FETP (SFETP) in enhancing the nation’s public health response, particularly amidst the tumultuous backdrop of armed conflicts that have left their indelible mark on the region.

**Methods:**

Employing a blend of quantitative and qualitative methodologies, we investigated the SFETP’s contributions to the public health response during the initial 4 months of the conflict (April–July 2023). Sixty-four SFETP residents and graduates were invited to participate, and data were gathered through semi-structured questionnaires.

**Results:**

A total of 44 (69%) SFETP residents and graduates were included in this study. Out of 38 SFETPs present in the states, 32 have considerably contributed to the crisis response at state and locality levels. Three-quarters of them have played key leadership, planning, and management roles. In essence, 38% (*n* = 12) of them have contributed to public health surveillance, particularly in data management, reports, Early Warning Alert and Response System (EWAR) establishment, and epidemic investigation. SFETPs have made special contributions to crisis response at the community level. The involved SFETPs supported WASH interventions (*n* = 4), and almost one-third of them strengthened risk communication and community engagement (*n* = 9). Despite their physical presence at the subnational level, 27% of graduates were not deployed to the crisis emergency response. Notably, throughout this time, half of the total SFETPs were formally retained during this response.

**Conclusion:**

The study highlighted the importance of FETP engagement and support during public health crises. SFETP residents and graduates played diverse roles in the various levels of public health emergency response to the crisis. However. Strategies to improve the deployment and retention of FETP residents are necessary to ensure their availability during crises. Overall, FETP has proven to be an asset in public health crisis management in Sudan.

## Introduction

On April 15, 2023, the armed conflict between the Sudanese Armed Forces (SAF) and the Rapid Support Forces (RSF) started in Khartoum state, Sudan. The conflict also extended to other states, and clashes between the two forces occurred mainly in the western part of the country, namely, North Kordofan State, North Darfur State, West Darfur State, and South Darfur State (1). By mid-August 2023, 6,277 injuries and 1,146 deaths were reported, mainly from Khartoum state (2). In addition, an estimated 968,451 persons crossed borders to other countries, including Ethiopia, Egypt, and Chad. A total of 3,282,303 million were internally displaced from states affected by the conflict; 72% were from Khartoum state. The northern, River Nile, and White Nile states harbor the most refugees (3, 4).

Preceding this turmoil, Sudan was already grappling with a series of disease outbreaks. Leading up to April 10, 2023, dengue fever emerged across 12 states, with Khartoum state harboring most of the cases, followed by North Darfur and Gedaref state (5). Multiple outbreaks also hindered the eradication or elimination of vaccine-preventable diseases. Circulating vaccine-derived poliovirus 2 (cVDPV2) outbreaks were declared on 18 December 2022, with one case related to a strain that circulated in Borno State, Nigeria (6, 7). In addition, Khartoum state and other states that are currently hosting internally displaced persons (IDPs) from Khartoum state were affected by large measles outbreaks (8). Furthermore, the current rainfall season, the poor environmental conditions surrounding the crisis, the massive population movement, and the interruption of curative and preventive health services are all red flags for new epidemics (9). Due to the conflict and poor environmental conditions, a cholera outbreak was reported from South Kordofan, in the western part of the country, and was associated with eight deaths. With massive war-associated population internal displacement, the outbreak extended geographically to eastern Sudan. The situation is further complicated by disrupted disease surveillance, public health emergency preparedness, and response, as well as the overall health system.

Effective preparedness, detection, investigation, and response to complex epidemiological situations demand a skilled and competent applied epidemiology workforce (10). As the demand for applied epidemiology capacity grew in the face of mounting public health threats, the Field Epidemiology Training Program (FETP) expanded globally, evolving into a three-tiered model. These tiers consist of the basic/frontline, intermediate, and advanced levels, tailored to support public health systems at the district, subnational, national, and regional levels (11, 12). This adaptable model caters to individual country and regional needs, focusing on core applied competencies such as epidemiology, data analysis, outbreak investigation, scientific communication (both verbal and written), surveillance evaluation, and public health leadership (13–15). Since its inception, skilled epidemiologists have played a crucial role in investigating outbreaks and establishing surveillance systems (11, 16). The FETP was established in Sudan in 2017 to build the capacity of epidemiologists to support different levels of the health system in the country; federal, state, or local levels. It aims to improve surveillance and public health response to outbreaks and emergencies. The training is predominantly field-based (75%), and only 25% didactic information in-class training. Two advanced FETP cohorts and one intermediate FETP cohort completed their training. In addition, two intermediate cohorts are currently running with 30 epidemiologists under training.

Despite over 6 years of establishment and investment in Sudan FETP (SFETP), no research has been conducted to assess its impact on public health response. The extent to which SFETP residents and graduates are effectively deployed, contributing, and meeting the country’s public health emergency needs remains unclear. This concern is heightened by the heightened demand resulting from the ongoing armed conflict crisis. Understanding the deployment status of SFETP residents and graduates in light of the crisis is crucial for effective planning and management. This study aimed to assess the pivotal role of the SFETP in enhancing the nation’s public health response, particularly amidst the tumultuous backdrop of armed conflicts that have left their indelible mark on the region.

## Methods

### Study setting and design

The SFETP operates under the Health Emergency and Epidemic Response Department within the Federal Ministry of Health. Our study employed a combination of quantitative and qualitative methods to assess the SFETP’s impact on public health response amid the initial 4 months of the conflict (April–July 2023). Utilizing the existing database, we invited all 64 participants, encompassing two cohorts of intermediate-level residents, one cohort of intermediate-level graduates, and two cohorts of advanced-level graduates, to participate in the study. We designed the study questionnaires using Google Forms and distributed the link through WhatsApp. Additionally, recognizing potential network constraints, we ensured accessibility by sharing the questionnaire via text message, enabling participants to seamlessly respond and return their inputs. To accommodate varied circumstances, we proactively conducted phone interviews, aiming to bolster response rates.

### Data collection

#### Questionnaire

A semi-structured questionnaire, comprising both open-ended and close-ended questions, was primarily used to determine whether residents and graduates experienced changes in their locations due to displacement, including potential relocation to other regions or departure from the country. The questionnaire consisted of 16 close-ended items covering demographic information, displacement status, pre-and post-conflict deployment, and an open-ended query exploring their contributions to the armed conflict emergency response.

#### Trainees’ field reports

We conducted an in-depth review of trainees’ field reports spanning the same time frame to identify prevalent themes highlighting the active engagement of residents in responding to the public health challenges posed by the armed conflict. These themes underwent a rigorous process of validation and corroboration, complementing and reinforcing insights gathered from the questionnaire responses.

#### SFETPs contribution form

Structured based on the identified thematic areas from the prior analysis, the “SFETPs Contribution Form” sought detailed information about participants’ roles in leadership, planning, and coordination of public health emergency responses during the crisis. It delved into their involvement in various aspects of public health surveillance (including data management, report writing, training officers, sustaining and managing surveillance), as well as their roles in outbreaks and case investigations. Additionally, the form inquired about their participation in needs assessments, risk communication, and community engagement activities.

### Ethical approval statement

The data utilized in this research constitute part of routine program monitoring data. Strict confidentiality measures were upheld throughout data handling, and the analysis was conducted anonymously.

### Data analysis

Data gathered from the three sources underwent compilation, thorough cleaning, and analysis using Microsoft Excel. Quantitative variables were assessed using frequencies and percentages. Open-ended responses underwent thematic analysis, subsequently cross-referenced with insights extracted from the review of residents’ field reports. ArcGIS, a geographic information system software, was utilized to visually illustrate the changes in SFETPs’ geographic distribution before and after the conflict, aiding comprehension.

## Results

### Impact of forced displacement on distribution of residents and graduates

Of the total 64 residents and graduates, 44 individuals (69%) responded to the questionnaire. Among respondents, nearly 65% were females (*n* = 29), with an average age of 38 (SD = 7) years. The distribution of SFETP residents and graduates was affected by forced displacements resulting from escalating clashes across various zones in the country and the capital state.

**Figure 1:**
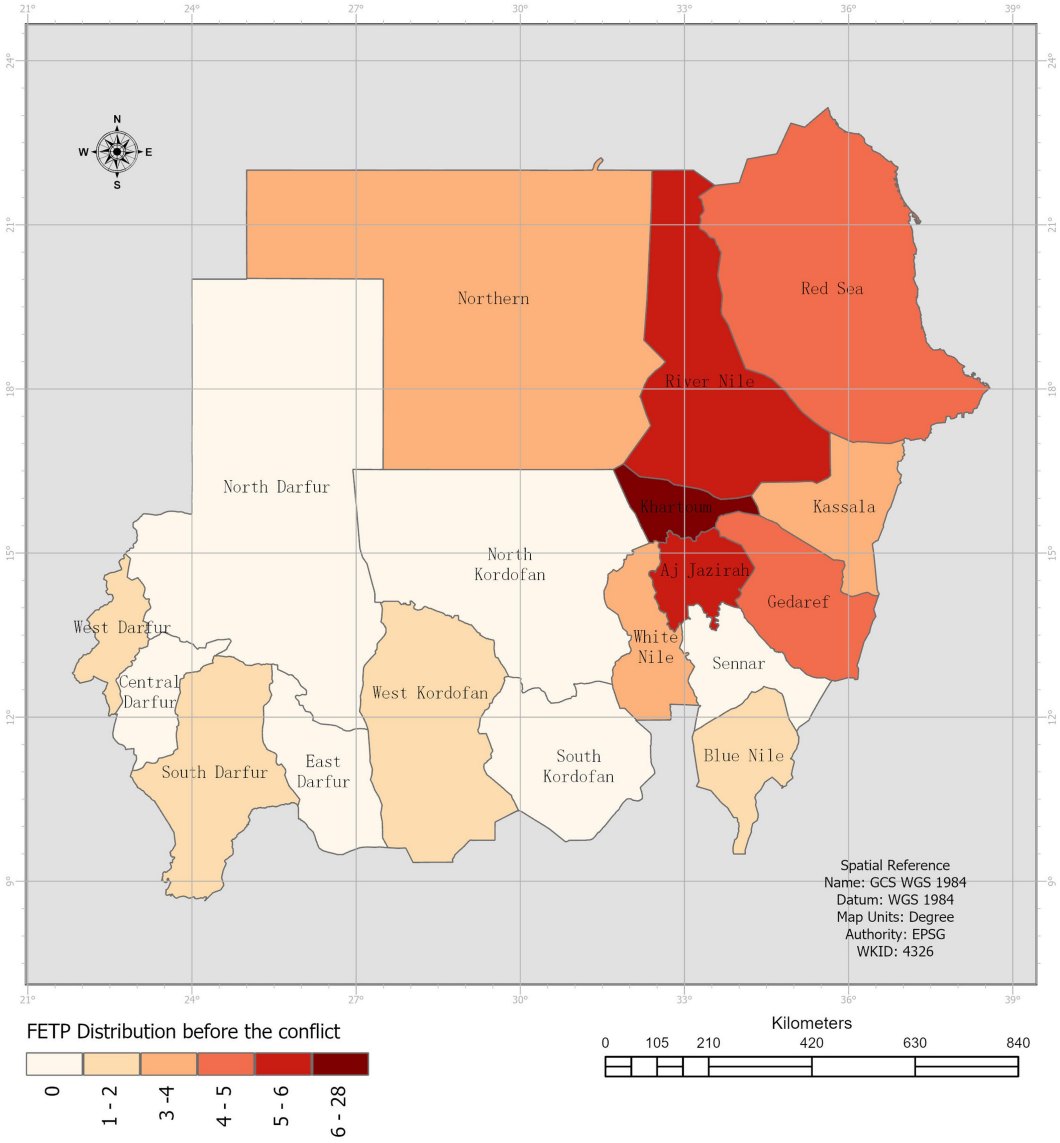
SFETPs Distribution before the conflict.

Before the conflict, residents and graduates were deployed in 67% (*n* = 12) of the states, increasing to 72% (*n* = 13) during the conflict. Notably, pre-conflict deployments were concentrated in Khartoum state, with 34% (*n* = 22) of the total. However, the conflict led to the displacement of SFETPs from Khartoum, south Darfur, and west Darfur states, with individuals relocating with their families to other states. Five individuals left the country, while four were displaced to Aj Jazirah state, approximately 72 miles from the capital. Notably, SFETP residents in West Darfur Ministry of Health fled to Chad due to the escalating conflict in the Geneina area (Figure 1).

**Figure 2:**
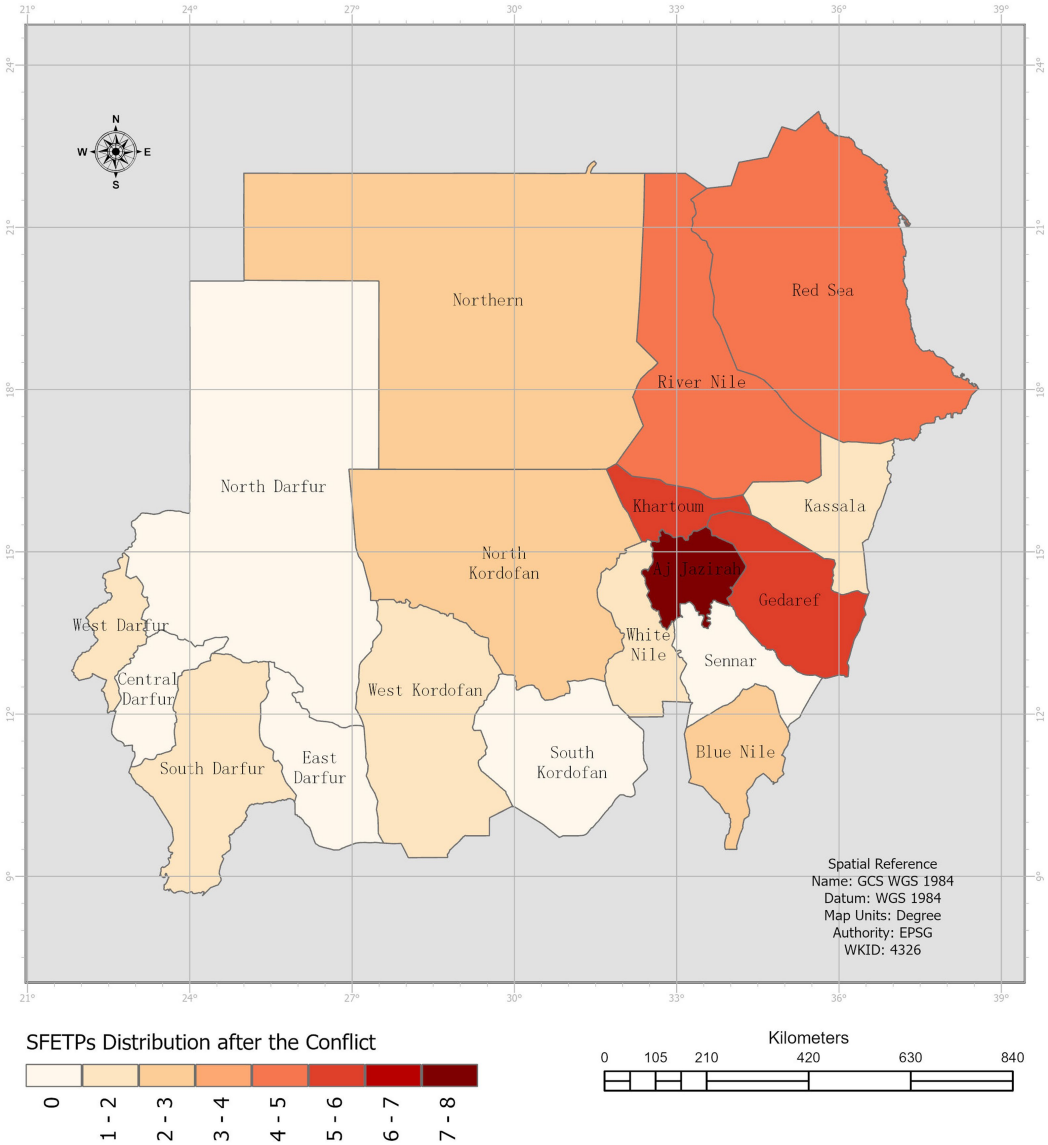
SFETPs Distribution after the conflict.

Following the conflict, residents and graduates were present in the central, eastern, and northern regions of the country, compared to their concentration in the capital state pre-conflict. Notably, North Kordofan state, previously without SFETP presence, hosted two displaced individuals from the federal level. However, there was a recognized need for increased presence in the western part of the country (Figure 2).

### Engagement of FETP residents and graduates in public health responses

During the conflict, 23 out of 30 residents and 15 out of 34 graduates were present. Among those present, 91% (*n* = 21) of residents and 73% (*n* = 11) of graduates were actively engaged in the response to the armed conflict (see Table 1).

**Table 1:** The FETP residents and graduates’ engagement in public health emergency response to the Armed conflict (per cohort), Sudan 2023.

Cohorts	Total	Present	Engaged in the response	Percentage of present who are involved in the response	Percentage of the total who are engaged in the response
	*N*	*n*	*n*		
*Residents*					
Second intermediate cohort (2022-now)	13	10	10	100%	77%
Third intermediate cohort (2022-now)	17	13	11	85%	65%
Total Residents	30	23	21	91%	70%
*Graduates*					
First intermediate Cohort (2021–2022)	11	4	3	75%	27%
First advanced cohort (2017–2019)	14	4	3	75%	21%
Second advance cohort (2020–2022)	9	7	5	71%	56%
Total graduates	34	15	11	73%	32%
Total	64	38	32	84%	50%

### Types of engagement

Leadership, Planning, and Coordination: Approximately 72% (*n* = 23) of SFETP residents and graduates played pivotal roles in leading and planning the public health emergency response during the crisis across state and locality levels (Table 2). These individuals undertook critical responsibilities such as leading Emergency Operation Centers (EOCs), overseeing surveillance departments, and strategizing mass casualty and dead body management in various regions.

**Table 2:** Types of SFETPs’ engagement in the public health emergency response to the armed conflict, Sudan 2023.

^a^Types of engagement (*N* = 32)	*n*	%
*Leadership, planning, and coordination*	23	72%
*Rapid needs assessments*	16	50%
*Elements of the assessments (N = 16)*		
Environmental	12	75%
Nutrition and food security	7	44%
Health status	11	69%
Demographics	5	31%
*Type of the assessments (N = 16)*		
Health facility	5	31%
Host community	4	25%
IDPs shelters	9	56%
*Health surveillance*	12	38%
*Areas of surveillance supported (N = 12)*		
Data management	8	67%
Report writing	6	50%
Training officers (locality, sentinel sites)	8	67%
Sustain surveillance	6	50%
Managing surveillance	3	25%
Outbreaks/case investigations	8	66%
*Risk communication and community engagement*	9	28%
*Service delivery*	3	9%
*Logistics and supply*	1	3%
*WASH*	4	13%

a SFETPs could be engaged in more than one area.

For instance, an SFETP resident assumed the lead public health officer role in Alfashaga Locality, Gedaref state, addressing the needs of approximately 1,000 Internally Displaced Persons (IDPs) who migrated from Khartoum state. The resident conducted comprehensive demographic and health profiling for IDPs in 14 host community neighborhoods and one IDP camp within a school, encompassing 3,477 individuals across 656 families. Moreover, this effort facilitated hygiene promotion, distribution of insecticide-treated bed nets, and vaccination record disaggregation for IDP households within the community.

Furthermore, SFETP residents and graduates actively engaged in coordinating with local NGOs and community members to promote hygiene practices, provide sanitary facilities, and conduct public health interventions, effectively addressing the needs of IDP populations and host communities.

Surveillance, Outbreak Investigations, and Response: More than one-third of residents and graduates (*n* = 12) supported surveillance activities, including managing surveillance data, writing epidemiological reports, and providing refresher training for surveillance officers at different levels (see Table 2).

Additionally, SFETP residents contributed to establishing an Early Warning, Alert, and Response System (EWARS) in collaboration with the International Organization of Migration (IOM) at entry points with Egypt. These efforts were crucial in strengthening health surveillance and response at the borders.

Moreover, 25% of the residents (*n* = 8) conducted outbreak investigations and responses, addressing acute watery diarrhea outbreaks among IDPs in various locations, including Gedaref state and cross-border regions of West Darfur and Chad.

Case Management and Rapid Needs Assessment: Half of the SFETP residents and graduates (*n* = 16) engaged in rapid needs assessments, focusing on IDP shelter needs, health, nutrition, and food security requirements (refer to Table 2).

Risk Communication and Community Engagement: Nine individuals (28%) actively contributed to risk communication and community engagement efforts in several states, promoting disease prevention measures and conducting educational sessions targeting community members.

Other Areas of Engagement: Thirteen percent of SFETP residents and graduates supported Water, Sanitation, and Hygiene (WASH) interventions, while others facilitated service delivery at entry points and provided logistics and supply support to health facilities (Table 2).

## Discussion

This study aimed to uncover how the SFETP supported the public health response during the initial 4 months of the country’s armed conflict crisis, described as the “worst humanitarian nightmare in recent history” (17). It revealed a noticeable shift in SFETP distribution pre-conflict due to forced displacement, especially affecting SFETPs in the capital and Darfur zones. Despite this upheaval, the majority of SFETPs who remained in the country (84%) contributed significantly across seven key areas such as leadership, planning, coordination, rapid needs assessments, health surveillance, risk communication, community engagement, service delivery, WASH, and logistics and supply.

The study highlighted a significant gap in SFETP distribution, deployment, and retention, with an uneven clustering of SFETPs even before the conflict, particularly favoring the central parts of the country over others like the Darfur and Kordofan zones. This disparity worsened with the conflict, concentrating SFETPs in the central state.

SFETPs were found in states directly or indirectly impacted by the conflict or influx of internally displaced individuals. However, despite many SFETPs being internally displaced and relocated to other states, formal deployment to support public health responses in their residing states was lacking. Consequently, 27% of mapped SFETP graduates were not contributing in their states, contrasting with over 90% engagement from residents. The disparity was attributed to the clear training pathways for residents, facilitating their deployment even during unstable times like crises, underscoring the need for better planning in deploying SFETP alumni.

Although almost 84% of mapped SFETPs supported the crisis public health response, this represented only half of the total SFETP graduates. Poor retention was evident, highlighted at the regional level by FETP advisors perceiving graduate retention as a crucial area for improvement (18), echoing a larger challenge in Human Resources for Health retention nationally within low-resource settings (19).

The SFETP made diverse contributions, primarily in three pivotal roles. Firstly, they excelled in leadership, planning, and coordinating crisis response efforts. This mirrors a comparable situation observed during Yemen’s conflict, where FETPs played a critical role in the COVID-19 response, focusing on emergency response planning and coordination (20).

The second significant role involved conducting rapid needs assessments, covering population demographics, health, nutrition, and the environment. Applied epidemiology, particularly in crisis response (21), allows for precise adaptation of humanitarian aid and public health interventions according to anticipated demographic and health changes during crises. This adaptation ensures effective targeting and addresses the actual post-crisis health priorities. Essential to these assessments were demographic surveys conducted at the administrative level, requiring a deep understanding of the local social, geographic, and political intricacies (22). Addressing these complexities is crucial to avoid inaccuracies in population size estimation (23). Epidemiologists well-versed in their communities can leverage these nuances to provide timely and relevant estimates that guide effective public health interventions. For instance, the FETP’s role in Gedaref state was noteworthy. Their deployment at the Al-Fashaga locality’s lowest administrative level enabled a thorough understanding of the social-cultural dynamics, revealing a tendency among IDPs to reside with their relatives (host community) rather than in camps. This insight is vital as both IDPs and host communities face negative health impacts from internal displacement. However, without reaching the host community, tracing these impacts becomes challenging. Through collaboration with community leaders, the FETP’s assessment unveiled additional estimates of IDPs within the host community, a crucial revelation that might otherwise have gone unnoticed.

The third crucial role encompassed supporting public health surveillance. While SFETPs have significantly contributed to routine activities such as data management and reporting, there is ample scope to enhance data utilization for decision-making. This includes conducting risk disease forecasting and assessments, vital components that should be explicitly integrated into current mapping efforts. In the face of disrupted surveillance systems, the ability to predict health outcomes through cumulative data analysis and cross-source integration holds immense potential to bolster preparedness and response to epidemics (24). Moreover, risk assessments should not solely focus on infectious diseases but should extend to encompass noncommunicable diseases, mental health, sexual, reproductive, and maternal health (25, 26). This comprehensive approach becomes pivotal in guiding effective and efficient health interventions during crises, ensuring a holistic response to diverse health needs.

While this study does not delve into assessing SFETP competencies, it’s essential to recognize the challenges crisis response poses, demanding a blend of sharp technical and nontechnical skills. The Ebola virus disease outbreak in West Africa from 2014 to 2015 highlighted specific skill gaps among epidemiologists, with about 25 to 30% reporting deficiencies in leadership, interpersonal communication, diplomacy, networking, and teamwork skills (27). Given the unique demands of crisis response scenarios, future studies should objectively evaluate and identify any training gaps faced by SFETP participants. This evaluation can pave the way for tailored additional training to equip them better for such exigencies.

### Study limitation

A significant limitation of this study was the low response rate among SFETPs, an expected challenge given the context in which the research was conducted. Respondents hailed from war-affected states, where pervasive poor internet connectivity posed a countrywide communication hurdle. To address this, diverse outreach strategies were employed, including phone calls and the option for respondents to reply via text message due to limited internet access.

## Conclusion and recommendations

The FETP emerged as a valuable asset in Sudan’s public health crisis management. However, gaps in SFETP task force management were evident, primarily concerning their uneven distribution, deployment, and inadequate retention. Thus, strategies aimed at enhancing the deployment and retention of FETP residents become imperative to ensure their availability during crises. Notably, SFETP residents and graduates exhibited leadership and technical prowess during the early phases of the armed conflict crisis response, showcasing significant impact at the local level. Future research should delve into assessing both technical and nontechnical gaps experienced during these critical experiences.

## Data availability statement

The original contributions presented in the study are included in the article/supplementary material, further inquiries can be directed to the corresponding author/s.

## Author contributions

AK: Conceptualization, Data curation, Formal analysis, Investigation, Methodology, Validation, Visualization, Writing – original draft, Writing – review & editing. HB: Writing – original draft, Writing – review & editing. HN: Writing – original draft, Formal analysis, Visualization. NA: Writing – review & editing, Methodology, Writing – original draft. YA: Writing – review & editing. AI: Writing – review & editing. SM: Writing – review & editing. MO: Writing – review & editing. YK: Conceptualization, Supervision, Writing – original draft, Writing – review & editing.

## Figures and Tables

**Table:** 

CDC	Center for Disease Prevention and Control
cVDPV2	Circulating Vaccines-Derived Polio Virus 2
EIS	Epidemic Intelligence Service
EMR	Eastern Mediterranean Region
EOC	Emergency Operation Center
EWARS	Early Warning Alert and Response System
FETP	Field Epidemiology Training Program
FMOH	Federal Ministry of Health
HRH	Human Resource for Health
IDPs	Internally Displaced Pearsons
IOM	International Organization of Migration
RRT	Rapid Response Team
RSF	Rapid Support Forces
SAF	Sudanese Armed Forces
SFETPs	Sudan Field Epidemiology Training Program residents
SFETP	Sudan Field Epidemiology Training Program residents and graduates
WASH	Water Sanitation and Hygiene
